# Thermography and thermoregulation of the face

**DOI:** 10.1186/1746-160X-3-17

**Published:** 2007-03-15

**Authors:** Jan Rustemeyer, Jürgen Radtke, Andreas Bremerich

**Affiliations:** 1 Department of Cranio-Maxillofacial Surgery, Klinikum Bremen Mitte, Bremen, Germany; 2 Department of Cranio-Maxillofacial Surgery, Universitätsklinik Knappschaftskrankenhaus Bochum-Langendreer, Bochum, Germany

## Abstract

**Background:**

Although clinical diagnosis of thermoregulation is gaining in importance there is no consistent evidence on the value of thermography of the facial region. In particular there are no reference values established with standardised methods.

**Methods:**

Skin temperatures were measured in the facial area at 32 fixed measuring sites in 26 health subjects (7–72 years) with the aid of a contact thermograph (Eidatherm). A total of 6 measurements were performed separately for the two sides of the face at intervals of equal lengths (4 hours) over a period of 24 hours. Thermoregulation was triggered by application of a cold stimulus in the region of the ipsilateral ear lobe.

**Results:**

Comparison of the sides revealed significant asymmetry of face temperature. The left side of the face showed a temperature that was on the average 0.1°C lower than on the right. No increase in temperature was found following application of the cold stimulus. However, a significant circadian rhythm with mean temperature differences of 0.7°C was observed.

**Conclusion:**

The results obtained should be seen as an initial basis for compiling an exact thermoprofile of the surface temperature of the facial region that takes into account the circadian rhythm, thus closing gaps in studies on physiological changes in the temperature of the skin of the face.

## Background

Within the framework of careful differential diagnosis of a wide range of different syndromes thermography has become increasingly important over the last few years since the introduction of the clinical diagnosis of thermoregulation by Schwamm^1^. Since it is simple and painless to perform and thus well accepted by patients, and its results are also well reproducible, thermography has become established an integral component of complex diagnostic schemata, especially in the diagnosis of pain and monitoring of the course of peripheral disorders of the cardiovascular circulation [2,3]. The diagnosis of thermoregulation is used on the one hand to determine the pattern of skin temperature in selected areas of the body during minimal stress to the regulation system [4,5], and on the other to record thermoregulation in these areas following the application of a defined cold stimulus [6,7], laser irradiation [8] or even acupuncture [9].

**Figure 1:**
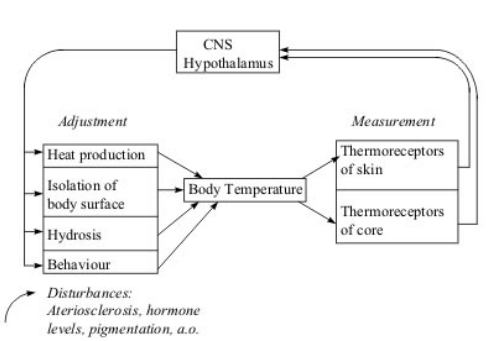
Adjustment of thermoregulation. The system contains 2 types of sensors, corresponding to the internal and external thermoreceptors of the organism.

Undisturbed central and peripheral regulation mechanisms that maintain a relatively constant core body temperature in dependence upon the environmental temperature are the basic precondition for intact thermoregulation (Fig. 1). The hypothalamus, which is the main organ involved in the integration of thermoregulation, processes information from external and internal thermoreceptors and leads to an adjustment of the actual and target temperatures [10-12]. Superimposed on this feed-back loop are phasic adjustments of the target temperature that occur in connection with changes in the circadian rhythm over the day and fluctuations in hormone levels [13]. The physiological skin temperature profile shows a temperature decline from the face through the abdomen to the feet, a relatively strict lateral symmetry of the two sides of the body and a distinct fall in temperature towards the distal portions of the extremities [6,14]. This temperature profile is subject to various influences, including arteriosclerosis, sympathetic tone [15,16], the heat and water metabolisms [17], the sudomotor system, the thickness and pigmentation of the skin and periodical fluctuations in hormone levels, e.g. the production of cortisol and progesterone [18,19]. While systematic research on the clinical thermography of the trunk and limbs has reached an advanced stage [14], the diagnostic value of thermography for the facial region must still be regarded as unclear. In order to be able to draw diagnostic conclusions on an adjustment of the target temperature by influencing the regulation system in the facial area, the circadian rhythm, uniform thermoregulation following application of a cold stimulus and a difference between values measured at symmetrically paired sites on the two sides of the face were investigated in healthy subjects as a reference group for further studies.

## Methods

### Subjects

14 female (aged 35,4 ± 12,4 y) and 12 male (aged 33,6 ± 13,7 y) subjects who had shown no history of previous diseases on a detailed questionnaire and did not meet the following generally recognised criteria for exclusion [20-22], were selected.

• previous diseases of the mouth, jaw or in the field of facial surgery

• acute inflammations of the upper respiratory tract

• facial cold injury or facial sunburn during the last 12 weeks

• dental treatment during the last 4 weeks

• pharmacotherapy with vasoactive substances or hormonal treatment including contraceptive pills

• a beard

• cosmetics in the face and neck area

• use of nicotine or alcohol on the day of measurement

• sports activities up to 2 hours before the measurement

### Conduct of the study

**Figure 2:**
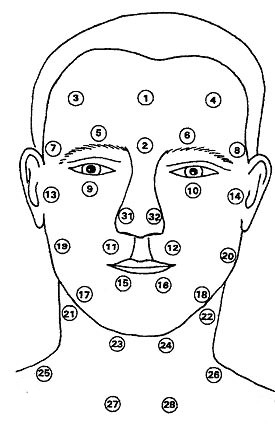
Measuring sites in the facial and neck regions and reference site (glabella) 1. Glabella. 2 Root of the nose. 3 Tabula frontale right (R). 4 Tabula frontale left (L). 5 Foramen supraorbitale R. 6 Foramen supraorbitale L. 7 Ramus temporalis R. 8 Ramus temporalis L. 9 Foramen infraorbitale R. 10 Foramen infraorbitale L. 11 Upper lip R. 12 Upper lip L. 13 Temporomandibular joint R. 14 Temporomandibular joint L. 15 Lower lip R. 16 Lower lip L. 17 Foramen mentale R. 18 Foramen mentale L. 19 Ramus massetericus R. 20 Ramus massetericus L. 21 Ramus submandibularis R. 22 Ramus submandibularis L. 23 Ramus submentalis R. 24 Ramus submentalis L. 25 Ramus supraclavicularis post. R. 26 R. supraclavicularis post. L. 27 Ramus supraclavicularis ant. R. 28 R. supraclavicularis ant. L. 31 Wing of the nose R. 32 Wing of the nose L. Measuring sites with identical regions of innervation: 1^st ^Ramus trigeminus R: 3,5. 2^nd ^Ramus trigeminus R: 7,9,11. 3^rd ^Ramus trigeminus R: 13,15,17. Plexus cervicalis R: 19, 21, 23, 25, 27. 1^st ^Ramus trigeminus L: 4,6. 2^nd ^Ramus trigeminus L: 8,10,12. 3^rd ^Ramus trigeminus L:14,16,18. Plexus cervicalis L: 20, 22, 24, 26, 28.

Thermographic investigations were carried out with an "Eidatherm" electronic contact thermometer (Werner Eidam, Medizin-Technologie GmbH, Albert-Boßler-Str. 2, D-35398 Gießen) and were performed by keeping standardized conditions given by previously released studies [21,22]: The measurements were commenced after a 15-minute adjustment period in a standardised room with a constant temperature of 22°C and a relative humidity of 50% and no additional radiators or direct sunlight. Dressings were consistent among subjects wearing T-shirts and jogging pants. The temperatures of the facial and neck areas were recorded by dermal contact with a nickel ring probe (2,5 mm radius, time response 0,1 sec) at 32 sites over a period of 24 hours. To ensure good reproducibility the sites selected were easy to find with reference to anatomic landmarks (Fig. 2). The measurements were taken at 4-hourly intervals at the following times: 2 a.m., 6 a.m., 10 a.m., 2 p.m., 6 p.m. and 10 p.m. For measurements at 2 a.m. and 6 a.m. no special arrangement was made, exceptional that subjects were awaken and transfered to the clinical centre. The thermographic device is designed in such a way that all measuring sites selected could be scanned within 60 seconds. The results were shown on a digital display. The device has an accuracy of ± 0.1°C specified by the manufacturer. After measurement of the glabella as reference value all further results were recorded as positive or negative deviations from the reference value. Mean values and standard deviations were calculated for comparison of the values obtained at different times of the day. The t-test for populations with a normal distribution was performed and 95% confidence intervals calculated to test for significance. Differences above the 95% confidence interval were regarded as statistically significant (p = 0,05).

To initiate thermoregulation for each series of measurements a brief, defined cold stimulus was applied by spraying both ear lobes with chloroethyl for 1 second [6,7].

## Results

**Figure 3:**
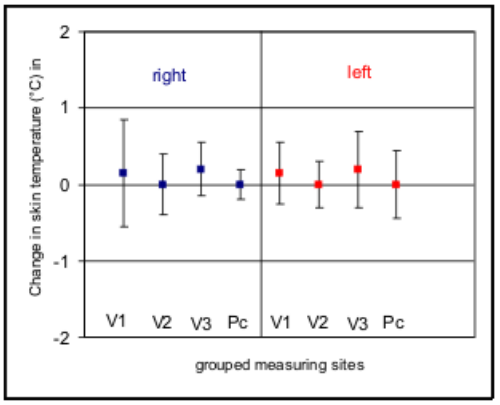
Means (lines) and standard deviations (bars) of temperature changes following application of a cold stimulus (n = 26). Isoline (0°) indicates mean data from the reference points (glabella). 32 measuring sites are grouped in 8 sites with identical regions of innervation in branches of the trigeminus nerve (V 1–3) and the cervical plexus (Pc) for the right and left side. Overall positive regulation in the areas of distribution of the 1^st ^and 3^rd ^branches of the trigeminus nerve on both sides, no regulation in the other areas. No significant thermoregulation in any area.

**Figure 4:**
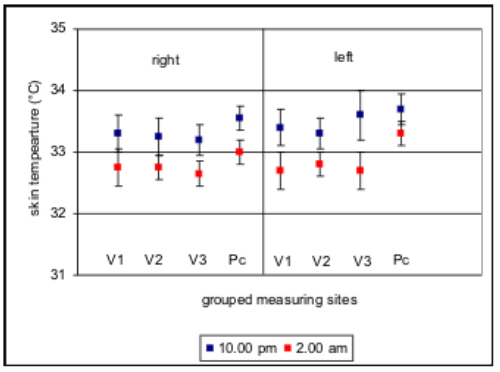
Means (lines) and 95% confidence intervals (bars) for facial skin temperature at the 8 grouped measuring sites at 2 a.m. and 10 p.m. as an example of a circadian rhythm of the facial skin. Abbreviations follow fig. 3.

**Figure 5:**
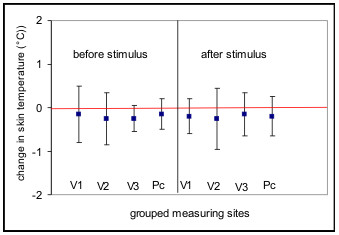
Comparison of the means (lines) and standard deviations (bars) of temperature differences between the right and left sides at the 8 grouped measuring sites before and after application of a cold stimulus for all measurements. References are the measurements of the right sides (red isoline). Abbreviations follow fig. 3. Temperature on the left side of the face being in a mean 0.1°C lower than that on the right side (p = 0,05), before and after application of the cold stimulus.

In the group of healthy subjects investigated no clearly significant thermoregulation was detected after application of the cold stimulus at any measuring time (p = 0,05). Even after grouping of the 32 measuring sites in 8 sites with identical regions of innervation in branches of the trigeminus nerve and the cervical plexus for the right and left side, the mean increase in temperature was only 0.1 C, and this increase was not detectable at all measuring times (Fig. 3). However, a significant circadian rhythm of the body surface temperature in the facial region was found. A circadian rhythm with a temperature minimum at between 2 a.m. and 6 a.m. and a maximum at between 6 p.m. and 10 p.m. was found across the 32 measuring sites both before and after application of the cold stimulus. The mean range of temperature fluctuation was 0.7°C. The greatest fluctuations in surface temperatures were found between the temperatures determined at 2 a.m. and 10 p.m. (Fig. 4). The changes in temperature following application of the cold stimulus in identical groups of measuring sites showed non-significant fluctuation ranges of up to 0.3°C and thus statistically no thermoregulation was found at individual measuring sites, even at different times of day. However, comparison of symmetrically paired values for the two sides revealed a distinct asymmetry (p = 0,05), the temperature on the left side of the face being in a mean 0.1°C lower than that on the right side. This asymmetry was evident both before and after application of the cold stimulus (Fig. 5).

## Discussion

While the circadian rhythm of the core body temperature is now probably one of the best investigated functions of the human body [23], to date there is no clear evidential basis on the circadian rhythm of the skin surface temperature in the facial region. Whereas muscular activity and ingestion of food were long considered to be decisive factors in the circadian fluctuations [24], these hypotheses have been disproved by various studies [25,26]. In his classification of temperature differences in the trunk and extremities before and after stimuli triggering thermoregulation, Rost [20] defined differences of 0.1–0.2 C as static temperature and 0.3–0.5 C as reduced temperature following thermoregulation. However, this was also not confirmed by further studies in the facial region. More recent results indicate that the extent of thermoregulation is far smaller, the differences ranging between 0.1 and 0.2 C [21,22].

In the present study particular consideration was given to the dependency of the surface temperatures and thermoregulation responses on the circadian rhythm. No clearly significant thermoregulation response to application of the cold stimulus was verified at any measuring time. The small changes in temperature detected in individual branches of the trigeminus nerve remained the same at all times of day, and thus the conduct of thermographic determinations at between 8 a.m. and 12 a.m. as recommended by Rost [20] failed to produce significant results. However, in healthy subjects increases in temperature of 0.1°C following application of a cold stimulus are only detectable in isolated cases, as demonstrated by Krischek-Bremerich and Bremerich [22]. Thus the significance of the small differences in temperature still classified by the above authors as thermoregulation should be reconsidered.

While some authors [23] still assume lateral symmetry of corresponding measuring sites, others [27-29] report temperature asymmetries of between 0.1°C and 0.3°C in the facial region which follow a regular pattern. This is consistent with our own observations, showing that left facial side temperatures are in a mean 0.1°C consistently lower than on the right side with no significance on subjects' ages. Irrespective of lateral asymmetry, in our subjects the skin surface temperature in the facial region showed a significant circadian rhythm under standardised conditions. The mean temperature difference of 0.7°C was only slightly below the 1°C mean fluctuation in the core temperature reported by Brück [30] and that of 1.2°C to 1.5°C found by Hensel [31]. The times at which the minimum and maximum temperatures were determined are also within the range demonstrated for the reference temperature, with a minimum in the early morning hours and a maximum in the evening.

## Conclusion

The results of this study in healthy subjects should prompt a reconsideration of the significance of thermographic diagnosis in the facial region. The next goal should be to establish an exact thermoprofile of the skin surface temperature in the facial region that takes into account the circadian rhythms, thus filling in the gap with regard to the body surface temperature left by studies on physiological changes in body temperature to date.

## Competing interests

The author(s) declare that they have no competing interests.
